# ePerturbDB: enhancer’s experimental perturbation database

**DOI:** 10.1093/database/baaf084

**Published:** 2026-01-15

**Authors:** Samiksha Maurya, Jaidev Sharma, Amit Mandoli, Vibhor Kumar

**Affiliations:** Department of Computational Biology, Indraprastha Institute of Information Technology, New Delhi 110020, India; Department of Computational Biology, Indraprastha Institute of Information Technology, New Delhi 110020, India; Department of Biotechnology, National Institute of Pharmaceutical Education and Research (NIPER) Ahmedabad, Palaj, Gandhinagar 382355, Gujarat, India; Department of Computational Biology, Indraprastha Institute of Information Technology, New Delhi 110020, India; Center of Excellence in Healthcare, Indraprastha Institute of Information Technology, New Delhi 110020, India

## Abstract

Enhancers act as *cis-*regulatory elements, controlling the expression of genes according to developmental stages, external signalling, and cell states. Recent studies have shown the impact of perturbation of enhancer activity on expression of genes and cell properties. However, at the same time, perturbation of many enhancers does not always show substantial effect on the expression of genes or properties of cells. Hence, there is a need to identify enhancers that can be effectively targeted for therapeutics and understanding regulation. Therefore, a comprehensive resource containing information on the effect of knockdown of enhancers is needed. Here, we introduce a database ePerturbDB, which provides resources to search the effects of 83 743 experimental perturbations of enhancers. The ePerturbDB database allows users to compare their genomic loci to the list of perturbed enhancers to know their potential effect. It also provides enriched genes and ontology terms for query enhancer location overlapping with a known experimentally perturbed enhancer list. Thus, the resource and tool in ePerturbDB can help users build hypotheses and design experiments to find effective enhancer-based therapeutics and inferences about the regulation of cell states. **Database URL**: http://reggen.iiitd.edu.in:1207/ePerturbDB-html/

## Introduction

Enhancers recruit transcription factors, co-factors, and chromatin modifier complexes to regulate transcription of genes. Enhancers are also known for affecting activity of genes according to species and cell states. Multiple methods have been proposed to identify active enhancers, which include biochemical assays involving sequencing to profile epigenome (ChIP-seq, DNAse-seq, and ATAC-seq) [1, 2], massively parallel reporter assay (MPRA) [3], luciferase-based assays, and mapping enhancer RNA (eRNA) [4]. While ChIP-seq of histone acetylation and P300 has been effective in identifying potentially active enhancers, the expression of bi-directionally transcribed eRNA at the location of enhancers is also associated with its activity. Several studies have also described priming and poising of enhancers before activation [5].

There are several databases that have experimentally validated or predicted enhancers, such as VISTA enhancer [6], SEA [7], dbSUPER [8], EnhancerAtlas [8, 9], and EnhancerDB [10]. While most of the enhancer databases are based on high-throughput assays like ChIP-seq and MPRA, a few databases also contain information about enhancers confirmed using low-throughput experiments. The FANTOM consortium has also published a list of active enhancers in different cell types determined using CAGE tag-based expression of eRNA [4]. The cancer eRNA atlas (TCEA) also provides a list of enhancers detected using eRNA expression in cancer samples.

While an enormous number of enhancers have been reported across different species, finding their effect and target is a challenging task. Several groups use the proximal genes as the most probable target of enhancers; however, it has been shown with chromatin interaction profiles that proximity does not confirm the connection between enhancers and promoters [11, 12]. Hence, assays such as HiC and HiChIP are often used to identify chromatin interactions between enhancers and promoters of genes. Analysis of single-cell ATAC-seq profiles [13] also helps to predict the interacting partner of enhancers [12]. However, due to several factors, even chromatin interaction profiles are insufficient to provide information about the direct effect of an enhancer perturbation on the guessed target promoter [11]. Therefore, researchers have been attempting to develop a computational method, such as the activity by contact (ABC) model, to predict enhancers that affect gene expression [11].

Multiple studies have employed the quantitative trait locus (QTL) approach to associate mutations in enhancers with gene expression. Few databases and resources provide predicted target genes of enhancers such as EnhancerAtlas, GeneHancer [14], and ENCODE-rE2G. GeneHancer provides a predicted link between enhancers and genes, where predictions were made using four different scores: tissue co-expression correlation between genes and eRNAs, enhancer-targeted transcription factors, expression quantitative trait loci (eQTL) within [11] enhancers, and capture Hi-C. ENCODE-rE2G used dataset of CRISPR-based knockout of enhancers in only the K562 cell line to predict enhancer–promoter interactions [15]. However, the predicted target of the enhancers may not always respond to perturbation of the enhancers [11]. Besides the effect on gene expression, researchers are also interested in the impact of target enhancers on the overall properties of cells. Therefore, several groups have performed experiments involving the perturbation of enhancers to study their effect on gene expression or cellular behaviour [16–18]. A few studies have also demonstrated the effect of eRNA knockdown on gene expression or cellular phenotypes [19]. More such experiments could be conducted in the future as enhancers are known to have better cell-type specificity in activity than promoters; therefore, they are considered safer targets for therapeutics. Given the fact that estimating the direct effect of enhancers in gene expression is not trivial, it could be even more challenging to estimate the effect of enhancers on cell phenotype. Hence, the eRNA and enhancer perturbation knowledge base is crucial for designing future experiments as well as therapeutic approaches targeting non-coding regions. To provide such a knowledge base, we have manually curated the results of enhancer and eRNA perturbation experiments in our database, ePerturbDB.

## Method and data collection

A comprehensive and systematic methodology was employed to collect, curate, and integrate diverse scientific literature alongside publicly available datasets focused on enhancer and eRNA knockdown/knockout studies. Enhancer annotations were meticulously gathered from multiple authoritative resources, including EnhancerAtlas, EnhancerDB [10], VISTA Enhancer Browser [8], and FANTOM database [4].

### Building the common enhancer list

Previously reported enhancer locations were downloaded from existing databases like FANTOM, VISTA, EnhancerDB, and EnhancerAtlas. Following preprocessing, extraction, and sorting of genomic coordinates, enhancer IDs (Enhrx) were designated for each enhancer from each database. The UCSC LiftOver tool was utilized to perform the coordinate conversion from hg19 to hg38. Bedtool [20] was employed to determine overlapping enhancer intervals between different datasets, after which all enhancers were combined into a single dataset with shared internal enhancer IDs while maintaining original database-specific IDs. Through this step, we systematically merged overlapping enhancer regions across the datasets, thereby creating continuous enhancer clusters. Each cluster was assigned a unique identifier (e.g. ‘enhrx-1’ and ‘enhrx-2’) for ease of reference, creating a common enhancer reference file.

### Literature mining and curation

The next critical phase involved an extensive literature search to identify experimental studies that reported enhancer or enhancer RNA (eRNA) knockdown or knockout experiments. The focus was placed on studies employing modern cell-perturbation screens, such as CRISPR-Cas9, CRISPR interference (CRISPRi), and RNA interference (siRNA and shRNA). Literature databases such as PubMed and Google Scholar were queried using relevant keywords, and selected research articles were manually reviewed to identify those involving the functional perturbation of enhancers or eRNAs.

Each selected article was thoroughly examined, with particular emphasis on the Methods section, to verify the experimental approach used for enhancer perturbation. The Data Availability sections and Supplementary Materials were carefully analysed to extract relevant information. Where available, we downloaded associated supplementary information or CRISPR screening datasets. For each enhancer perturbation experiment, detailed metadata were curated, including the chromosomal location, genomic coordinates, genome version, perturbation method (e.g. sgRNA, shRNA, and siRNA), and the type of enhancer (enhancer/eRNA). We also extracted contextual biological information such as the cell line and tissue type used in the study, the reported knockout effect (significant or non-significant), and whether the observed effect was direct or indirect with respect to target gene regulation.

In cases where only the targeting sequences (e.g. sgRNA or shRNA) were reported and the corresponding genomic locations were not provided, we used the BLAT (BLAST-like Alignment Tool) from the UCSC genome browser to map these sequences to the human genome, allowing to determine the precise genomic coordinates of the targeted enhancer loci. Once all enhancer perturbation entries were curated and mapped, the coordinates were again standardized to the hg38 genome version using the LiftOver tool, ensuring consistent genomic reference across the entire dataset.

### Enhancer-to-gene association and functional annotation

With the curated enhancer regions mapped to hg38, we aimed to associate each enhancer with its nearest gene, for understanding the regulatory consequences of enhancer perturbations. We employed the bedtool closest function to achieve this, which computes the nearest genomic feature (e.g. a gene) for a given interval. Gene annotations from the Ensembl database served as the reference for determining gene coordinates and transcription start sites (TSS), and the associated gene name, coordinates, and chromosome were used. This mapping provided insights into potential enhancer–gene regulatory relationships. In addition to gene association, we annotated each enhancer with basic genomic properties, such as its width (in base pairs) and distance from the enhancer to TSS, to help in evaluating potential enhancer strength and functional significance.

### Data integration with reference enhancer set

After completing the curation of literature-derived enhancer perturbations, we compiled a total of 38 266 unique enhancer perturbation entries. Each entry was assigned a unique internal identifier for tracking and merging purposes. The curated enhancer coordinates were intersected with the previously constructed common enhancer reference file (from EnhancerAtlas, FANTOM, VISTA, and EnhancerDB). This intersection step was performed using bedtool, which allowed the identification of curated enhancer loci that overlapped with known enhancers in established databases. Through this intersection, we obtained a combined set of 75 977 enhancer entries, which were then merged with the original curated enhancer data using internal IDs. This final integration step resulted in a total of 83 744 enhancer perturbation records.

Each entry in the final enhancer perturbation database includes a rich set of metadata fields, such as enhancer ID, chromosome, start and end coordinates, genome version, database-specific IDs, internal keys, enhancer classification (enhancer or eRNA), sgRNA, shRNA, and siRNA sequences, and knockout effect. Additional fields include target classification, cell line, tissue type, and gene annotations (both literature-reported and nearest gene). Where available, we included statistical metrics from the original studies, such as Z-scores, log2 fold change (log2FC), beta coefficients, *P*-values, and CRISPRi scores. Provenance information—such as paper titles, DOIs, PMIDs, and assigned enhancer groups—was also recorded for traceability.

Rigorous quality control measures were implemented to remove duplicates and resolve metadata discrepancies, culminating in a unified, robust database that integrates both computationally derived enhancer annotations and manually curated knockout data.

### Final database utility

**Figure 1.**
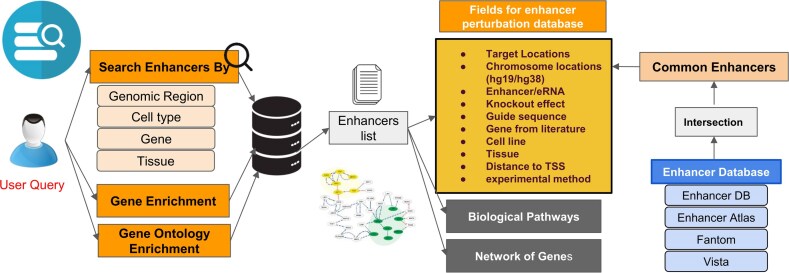
Overview of ePerturbDB website. Users can search the database using genomic region, cell type, gene, or tissue name. ePerturbDB provides results in terms of matching enhancer locations. For each matching enhancer, it provides other details such as study ID, protocol, and corresponding CRISPR sgRNA or siRNA used to perturb it experimentally. It also provides enriched gene ontology and genes for matching enhancers in its database.

The final enhancer knockout database, ePerturbDB, represents a comprehensive integration of manually curated literature data and computationally standardized enhancer annotations. Thus, by leveraging both experimental and public domain resources, this database captures the functional landscape of enhancer perturbations in human biology. Each enhancer entry is anchored to genomic coordinates (in hg38), mapped to known enhancers, annotated with gene proximity data, and enriched with biological and experimental context. The framework of the ePerturbDB web portal has REST Api-based functions for scalability. The curated database is suitable for various downstream analyses, such as classification of enhancers/eRNAs, regulatory effect studies, enhancer–gene network construction, and functional genomics investigations (see Fig. 1).

### Estimating enrichment score of genes and gene ontology

To estimate the enrichment score of genes according to their frequency around a set of enhancers, we made a list of genes for every enhancer in the foreground and background list. The foreground list of enhancers is provided by the user. The background list consists of all enhancers curated in ePerturbDB and the foreground list. Notice that the list of nearest genes would also have repetitions; hence, we counted their frequency. Using the proximal genes to enhancers in the foreground and background list, we calculated the significance of the enrichment of genes using a hypergeometric test, as described in Mishra *et al*. [21]. For estimating the *P*-value of enrichment of gene ontology terms, we applied the same approach as the GREAT GO tool [22]. For pathway enrichment, we have used gene sets from the list of GO Biological Process 2025, KEGG 2021 Human, Reactome Pathways 2024, WikiPathways 2024 Human, and Human Disease Signatures Database curated from EnrichR webserver (https://maayanlab.cloud/Enrichr/).

## Results and discussion

With extensive manual curation and systematic integration, the final database comprises approximately 83 743 enhancer knockout/knockdown records. This resource includes detailed genomic coordinates, unique enhancer IDs, cross-database identifiers, gene associations, and experimental metadata related to knockout effects. The consolidation of data from multiple sources has enabled the identification of overlapping enhancer regions and the comprehensive documentation of enhancer knockout effects, providing critical insights into enhancer functionality and gene regulation.

### Features and functionality

**Figure 2.**
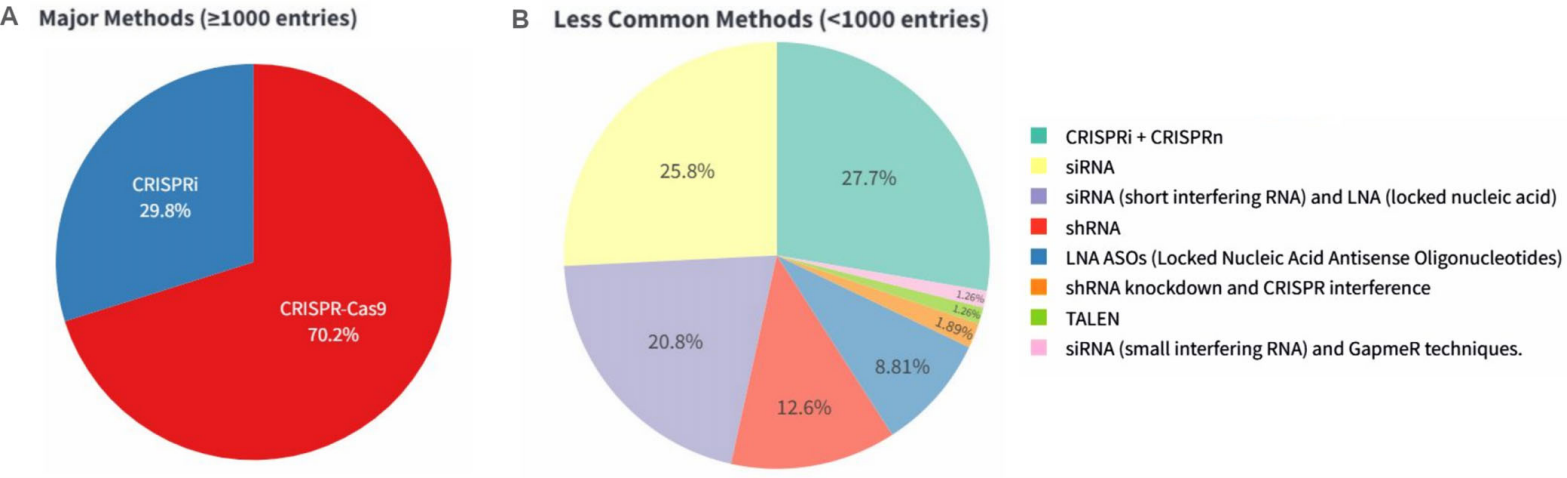
Distribution of perturbed enhancer loci and eRNA according to experimental method. (A) Pie chart showing the fraction of enhancer perturbations in ePerturbDB, which are based on major methods (CRISPR-Cas9 and CRISPRi). (B) Pie chart showing the distribution of enhancer or eRNA perturbation (in ePerturbDB) based on less commonly used methods.

The database features detailed annotations for each enhancer, including chromosomal coordinates, unique enhancer IDs, and database-specific identifiers. It integrates various knockout/knockdown methodologies (Fig. 2) such as CRISPR-Cas9 (including sgRNA)–58 646, CRISPRi (including sgRNA)–24 982, siRNA–76, shRNA–23, LNA-ASOs (locked nucleic acid antisense oligonucleotide)–14 and, TALEN-mediated–2 (see Fig. 2) along with corresponding experimental details like target genes, cell lines, tissue types, and observed phenotypic outcomes. The curated information on knockout/knockdown experiments involving enhancers and eRNA is derived from more than 29 cell lines and 21 tissue types. This integration allows for a multifaceted exploration of enhancer functionality, regulatory mechanisms, and their impact on gene expression across diverse biological contexts.

### Web interface

A user-friendly web interface has been developed to facilitate seamless access to the database. The interface allows users to browse, filter, and visualize data through integrative genomic maps and customisable search options. Detailed annotations and direct links to source publications (including DOIs and PMIDs) are provided for each record, ensuring that users can easily retrieve relevant experimental context and validate findings (see Fig. 1).

### Search for information

Robust search functionality is a key feature of the database. Users can query enhancer knockout records by enhancer ID, gene name, chromosomal location, or experimental method. Advanced filtering options enable further refinement of searches based on criteria such as cell type, knockout method, or observed effects, ensuring that researchers can quickly and efficiently access the most pertinent data. This comprehensive search capability makes the database an invaluable tool for studying enhancer regulation, gene expression dynamics, and the broader implications of enhancer perturbations. Thus, by combining our in-house data integration approach with exhaustive literature curation, this database serves as a valuable resource for the research community to investigate enhancer functionality and its impact on gene regulation.

**Figure 3.**
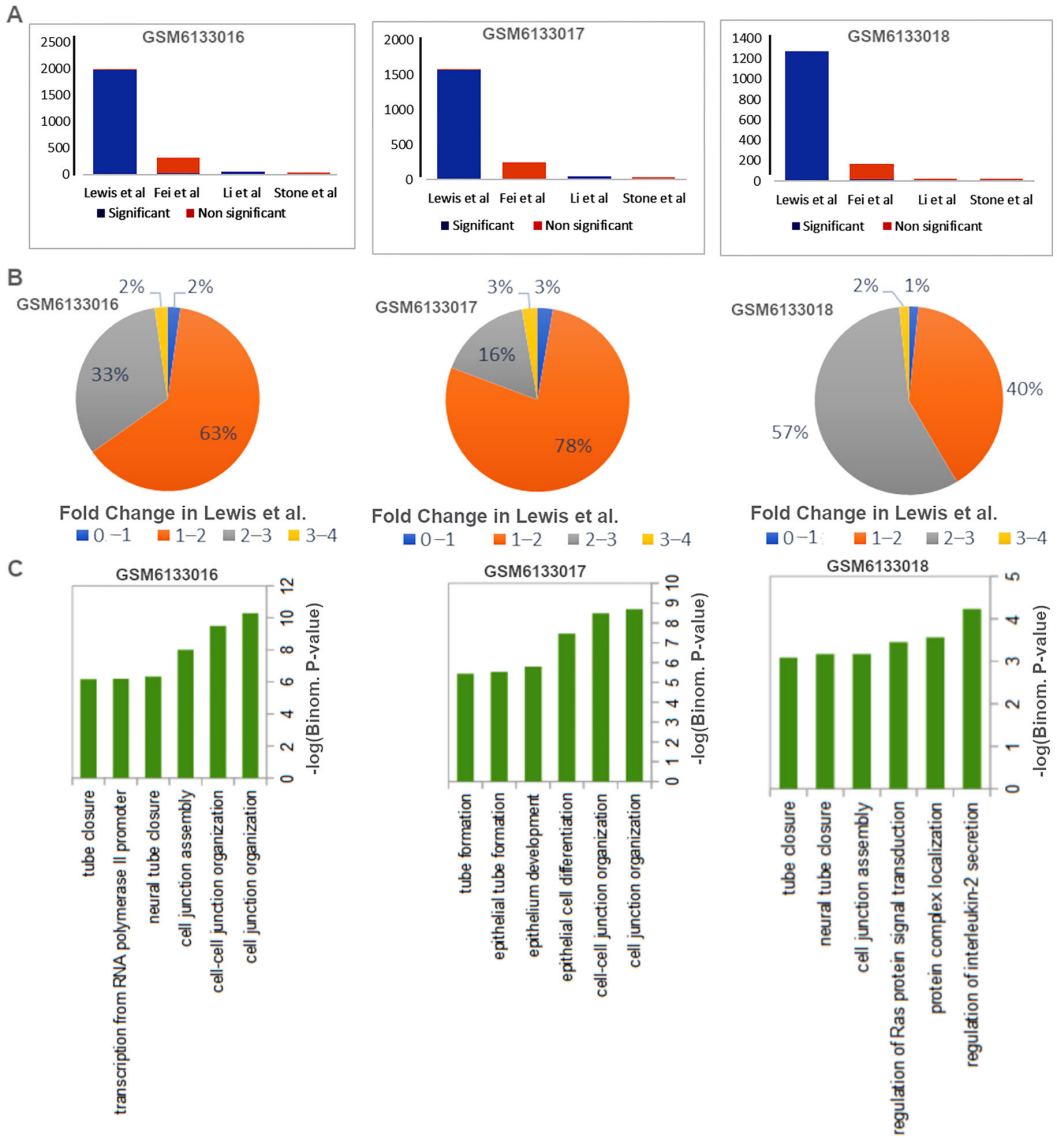
A case study of finding overlap of experimentally perturbed enhancers with predicted enhancers using H3K27ac ChIP-seq peaks from patient-derived xenograft (PDX) models of triple-negative breast cancer (TNBC). (A) Bar plot showing the number of experimental perturbations (sgRNA or eRNA-based knockout or knockdown) in breast cancer cell lines from different studies overlapping with predicted enhancers in the cell line model for three TNBC patients. (B) Pie charts showing the distribution of reported fold changes of target genes by experimental perturbation of enhancers or eRNA reported by Lewis *et al*. [16] and overlapping with predicted enhancers. (C) Enriched biological process for proximal genes to enhancers, which were perturbed in breast cancer cell lines in four different studies and overlapped with H3K27ac peaks from the PDX model of three TNBC patients.

**Case study 1:** In order to demonstrate the utility of ePerturbDB in finding potential targets in cancer cells, we tested it on enhancers predicted using peaks of H3K27ac ChIP-seq of the PDX model for tumours from triple-negative breast cancer (TNBC) patients, published by Jovanovic *et al*. [23]. We submitted a list of peaks of H3K27ac ChIP-seq for the PDX model of tumours from three TNBC patients to the ePerturbDB query page. It provided a list of the overlapping enhancers and eRNA and their corresponding sgRNA and siRNA used for perturbing their activity. For H3K27ac peaks from the PDX model named 4272-TG5 (GEO ID: GSM6133016), we got 321 overlapping enhancers from four studies that were perturbed in different breast cancer cell lines. For these 321 enhancers, a total of 2299 sgRNAs were used either for CRISPR-Cas9 or CRISPRi-based perturbation by Fei *et al*. [24] and Lewis *et al*. [16], respectively. Our database also provided three siRNA target locations that were previously used to knock down eRNAs, overlapping with H3K27ac peaks from the 4272-TG5 PDX model. Those three siRNAs were used by Li *et al*. [25] and Stone *et al*. [19]. The data in ePerturbDB revealed that Lewis *et al*. had reported significant effects in the MDA-MB-436 cell line for most of the sgRNAs used for CRISPRi of sites overlapping with enhancers in the TNBC PDX model (see Fig. 3B). Such as for enhancers in PDX models 4272-TG5 (GEO ID: GSM6133016), 5998-TG5 (GEO ID: GSM6133017), and BCM-3107 (GEO ID: GSM6133018), respectively, 37%, 19%, and 59% of overlapping CRISPRi sgRNAs from Lewis *et al*. have an effect more than two-fold change in target gene expression in MDA-MB-436 cell lines. The gene ontology enrichment analysis using GREAT GO tools [22] revealed that enhancers from PDX models of TNBC for which we found overlapping experimental perturbation reports mostly had top functional terms related to cell junction organization, tube formation, and epithelium development. Tube formation property of TNBC cells is well known [26]; however, the information about effective guide RNAs to target it through CRISPRi in the PDX model of clinical TNBC sample is valuable for oncology. Thus, ePerturbDB provides a valuable resource for estimating the effect of targeting enhancers in patient-specific cancer models.

**Figure 4.**
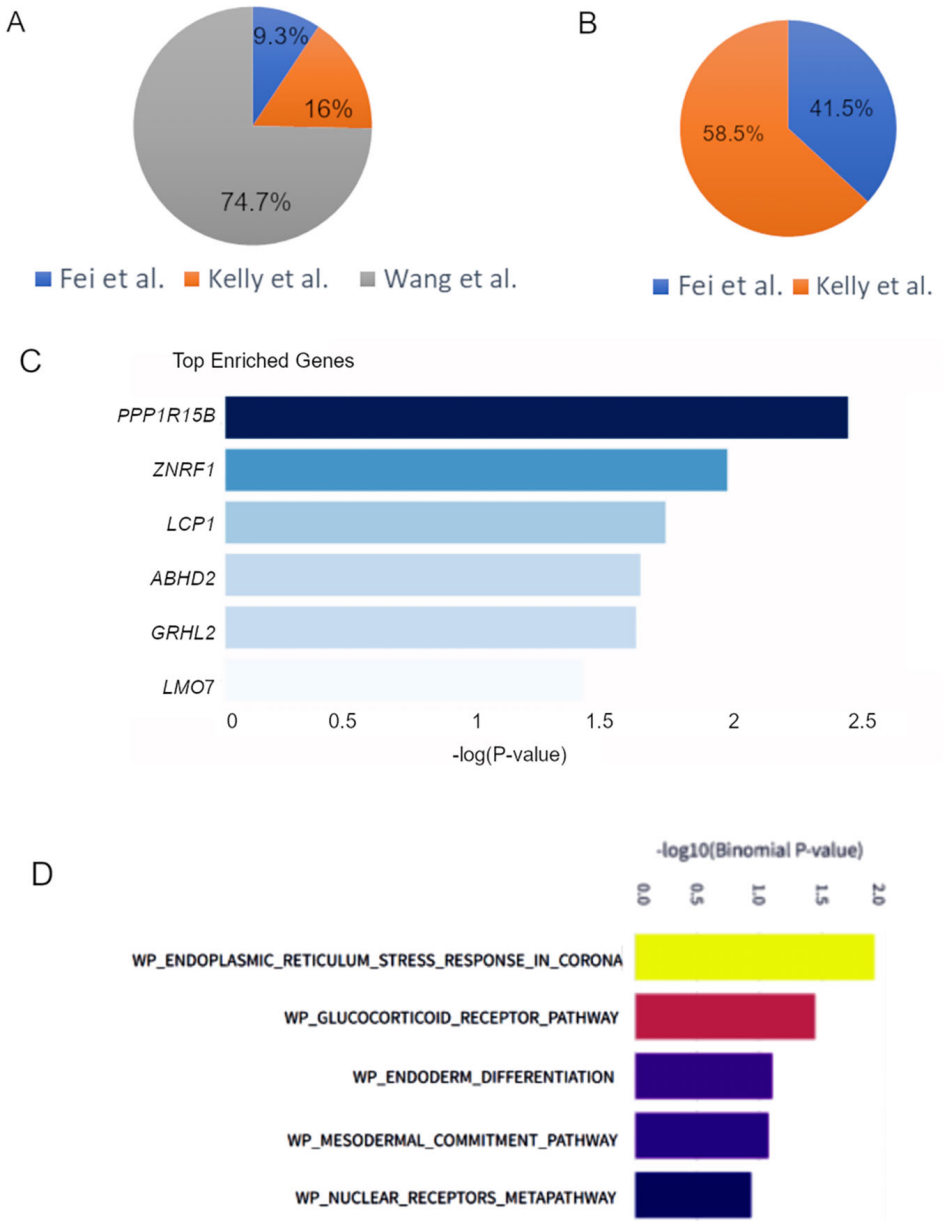
An overview of the results of the intersection of eRNA associated with survival in breast cancer (in TCEA database) with the enhancer perturbations curated in our database. (A) Distribution of intersecting enhancer perturbations in terms of the source of study. The overlapping enhancer perturbations with TCEA-BRCA-eRNA mainly came from three different studies (Fei *et al*. [24], Kelly *et al*. [28], and Wang *et al*. [27]). (B) The distribution of the source of overlapping enhancer perturbations, which showed a significant effect in the corresponding study. (C) Top enriched genes around perturbed enhancers overlapping with TCEA-BRCA-eRNA. (D) Top enriched gene ontology for enhancer locations in ePerturbDB overlapping with TCEA-BRCA-eRNA.

**Case study 2:** To demonstrate the utility of our database, we tested it using 327 eRNA locations reported in the TCEA database as significantly associated with breast cancer survival in the TCGA (TCEA-BRCA-eRNA) cohort. Within 500 base pairs of 9 eRNA loci, we identified 75 overlapping gRNA locations in our database, which were used for either CRISPR-Cas9 or CRISPRi-based perturbation of enhancers in three different studies. Out of 75 sgRNAs, 56 (∼74.7%) came from the study of Wang *et al*. [27]. However, none of them were reported to have a significant effect by the authors (Fig. 4A). The overlapping sgRNAs with significant effects were reported by Fei *et al*. [24] and Kelly *et al*. [28] (Fig. 4B). The ePerturbDB also provided an indication of the significance of gene enrichment around eRNAs that overlapped perturbation sites (Fig. 4C). The most enriched gene around enhancers in ePerturbDB, overlapping with TCEA breast cancer eRNA locations, was *PPP1R15B*. Similarly, ePerturbDB also provided a *P*-value for the enrichment of gene ontology terms for enhancers (eRNAs) with overlapping perturbed sites.

## Future prospect

In future, we will add more datasets of perturbed enhancers and eRNA as they get published. We would also attempt to link the curated enhancers with multi-omics datasets and provide users with additional functionality for comprehensive analysis. We look forward to assisting researchers in understanding the role of enhancers and targeting them for therapeutic purposes.

## Data Availability

The ePerturbDB database is freely available through a web link http://reggen.iiitd.edu.in:1207/ePerturbDB-html/. User can also use the link http://reggen.iiitd.edu.in:1207/ePerturbDB-html/downloads/enhancers_details_simple.bed to upload as a custom track in the UCSC genome browser. It does not require any registration or login access for using. In addition to online services on the database portal, the data can be downloaded freely.
